# Investigating PAK inhibition in combination with PD-1 blockade to enhance cytotoxic CD8+ T cell-mediated killing and suppress invasion of ovarian cancer cells

**DOI:** 10.1038/s41416-026-03342-z

**Published:** 2026-03-06

**Authors:** Alexandria R. Mitchell, Yiran Chen, Giuseppe Pugliese, Gabriela Vino Flores, David M. Davies, John Maher, Claire M. Wells

**Affiliations:** 1 Comprehensive Cancer Centre, King’s College London, London, UK; 2 Leucid Bio, London, UK; 3 Department of Immunology, Eastbourne Hospital, Eastbourne, UK

**Keywords:** Cancer, Ovarian cancer

## Abstract

**Background:**

P-21 activated kinase (PAK) overexpression, phosphorylation, and gene amplification have been reported to increase cellular invasion in ovarian cancer (ovcan), worsening patient prognoses. One notable method of ovcan survival is through the PD-(L)1 checkpoint pathway, and PD-L1 expression in ovcan is correlated with poor patient outcomes. However, PD-1 and PD-L1 targeted clinical trials in ovcan have shown modest results. This work has examined the possibility of using PAKi and PD-1 blockade as a combination therapy.

**Methods:**

PAK and PD-L1 expression in ovarian cells was determined. A novel 3D spheroid assay was used to assess ovcan invasion. Ovcan cell viability, downstream pathway signalling, and surface PD-L1 expression were evaluated after treatment with PAK inhibitors and co-culture with cytotoxic CD8^+^ T cells. Ovcan cell and CD8^+^ T cell co-cultures were treated with a combination of PAK inhibition and PD-1 checkpoint blockade and ovcan cell viability was assessed.

**Results:**

Ovcan cells showed significant sensitivity to PAKi. CD8^+^ T cell killing of ovcan cells improved following pre-treatment with PAK inhibitors, and this was further augmented with PD-1 blockade.

**Discussion:**

The work presented here demonstrates the efficacy of PAK inhibition and PD-1 checkpoint blockade as a combination therapy for high-grade serous ovarian cancer.

## Background

Ovarian cancer (ovcan) is the sixth most common cause of cancer death among women in the United Kingdom [1] and is the most lethal gynaecological cancer [2]. More than 90% of patients are diagnosed with high-grade serous ovarian cancer (HGSC), a subtype of epithelial ovarian cancer (EOC). HGSC is characterised by an aggressive phenotype, poor patient prognoses, and metastasis [3]. There are no reliable screening measures for ovcan, where symptoms have physical manifestations but are non-specific. Current treatments are based on chemotherapy and debulking surgery, and diagnoses are often made at an advanced stage when treatment options for HGSC are limited, and metastasis has occurred.

P-21 activated kinase (PAK) overexpression, phosphorylation, and gene amplification have all been reported in ovcan [4], accompanied by altered cellular proliferation, migration and patient outcomes. PAK kinases are subdivided into Group I (PAK1-3) and Group II (PAK4-6) [5]. PAK4 is the most extensively studied family member in ovcan, and previous work strongly suggests that overexpression of PAK4 and its activated, phosphorylated form p-PAK4 correlate with progression and poor prognoses of ovcan patients [6]. Additionally, in vitro and in vivo studies suggest that PAK4-enhanced proliferation of SKOV3 cells and PAK4 regulation of ovcan cellular migration and invasion occurs by way of two downstream pathways: PAK4/c-Src and PAK4/MEK1/ERK1/2 to MMP2 [7]. PAK1 is also overexpressed in ovcan, and both PAK1 and PAK2 have been proposed as potential molecular targets for ovcan therapy [8, 9]. Knockdown of PAK1 and PAK2 in ovcan cell lines reduced their migration and invasion [10], and when PAK1 is phosphorylated, it promotes epithelial to mesenchymal transition (EMT) in ovcan cells.

Several PAK inhibitors have been developed as therapeutic agents. Small molecule inhibitors targeting PAK activity decrease proliferation and invasion of several cancer types, including breast, thyroid, pancreatic, and prostate [11–13]. A majority of PAK inhibition (PAKi) studies focus primarily on PAK1 inhibition as it is overexpressed in several cancer types [4]. PAK1 inhibition was reported to facilitate anti-tumour immunity in pancreatic ductal adenocarcinoma (PDA) [14]. Group I PAKi has also been reported to significantly decrease downstream ERK phosphorylation activity, proliferation, and tumour weight in ovcan in vivo xenografts [15]. Most recently, inhibition of a RAC1/PAK4/MAPK pathway has also been shown to reverse ovcan paclitaxel resistance in mouse tumour models and in clinical samples [16]. Despite the undeniable role of PAKs in ovcan progression, invasion, and patient prognoses [6, 9, 10], there has been little exploration of the effects of PAKi on downstream phosphorylation activity or invasion in HGSC specifically [9, 15]. While PAKi studies in ovcan thus far have been promising, results suggest that PAKi in combination with another therapeutic target could further improve treatment outcomes [17].

PD-L1 expression is reported in several cancers, including melanoma, ovarian, oesophageal, stomach, breast, and kidney, and high expression in ovcan is significantly positively correlated with poor patient outcomes [18]. A significant inverse relationship between PD-L1 expression and CD8^+^ TIL count suggests that PD-L1 expression in ovcan is a noteworthy prognostic factor [18]. and PD-L1 expression in ovcan coincides with infiltration of cytotoxic CD8^+^ T cells [19]. However, anti-PD-L1 therapy in a phase 1 clinical trial using avelumab in advanced ovarian cancer patients achieved only modest results [20]. Patients had previously treated (chemotherapy) recurrent or refractory ovarian cancer, and the 1-year progression-free survival rate was 10.2% (95% CI, 5.4–16.7%), and the median overall survival was 11.2 months (95% CI, 8.7–15.4 months) [20]. Similarly, anti-PD-1 blockade using pembrolizumab also demonstrated modest results [21]. In the pembrolizumab phase 2 KEYNOTE 100 clinical trial, patients with advanced recurrent ovarian cancer had an overall response rate of 8% and progression-free survival was 2.1 months [21]. These studies raise the possibility that baseline immunophenotypic screening in ovcan, followed by PD-(L)1 in combination with other biological agents, may be more beneficial compared with a monotherapy where efficacy is poor [21–23].

This study has investigated whether a combination of PAK inhibition and PD-1 checkpoint blockade could limit HGSC cell invasion and dissemination and bolster cytotoxic CD8^+^ T cell-mediated killing of these cells.

## Methods

### Ovarian cancer cell lines and cell culture

SKOV-3, OVCAR-3, and OVCA433 cell lines were purchased from the American Type Culture Collection (ATCC, VA, USA), STR validated. IGROV1 cells were kindly provided by Philip Blower at King’s College London. A2780 and CP70 cell lines were kindly provided by Bob Brown at Imperial College London. The Ovsaho cell line was kindly provided by John Maher at Guy’s Hospital, and the Kuramochi cells were kindly provided by Patrick Caswell from the University of Manchester. SKOV-3, IGROV1, Kuramochi, A2780, and CP70 were cultured in RPMI-1640 (Sigma) supplemented with 10% heat inactivated fetal bovine serum (FBS; Gibco), and 1 mM penicillin–streptomycin (Gibco). OVCAR-3 cells were cultured in RPMI-1640 supplemented with 20% FBS and 1 mM penicillin–streptomycin. OVCA433 and Ovsaho cells were cultured in DMEM (Sigma) supplemented with 10% FBS and 1 mM penicillin–streptomycin. Cells were maintained in a humidified atmosphere containing 5% CO_2_ at 37 °C. All cells were routinely checked for mycoplasma

### PAK inhibitors

Cancer Research UK (CRUK) provided all PAK inhibitors under a material transfer agreement. The CRUK-designed Pan-PAK inhibitor is referred to here as “Pan PAKi” [24]. The CRUK formulated Group II inhibitor [Compound 31] [25]. is referred to as “Group II PAKi” [Compound 31 *K*_i_ of 3.112 μM for PAK1 and *K*_i_ of 0.009 μM for PAK4]. Finally, the CRUK formulated Group I inhibitor [G-5555] [26] is referred to as “Group I PAKi” [G-5555 *K*_i_ of 3.7 nM for PAK1 and 11 nM for PAK2].

### PD-1 blockade

Pembrolizumab (Keytruda) was provided by Dr. John Maher at Guy’s Hospital.

### Immunoblotting

Cell lysates were separated by acrylamide gel electrophoresis. After separation, proteins were transferred onto a nitrocellulose membrane for overnight incubation at 4 °C with primary antibodies (Table 1). Membranes were washed in TBST and incubated at RT with respective HRP-conjugated secondary antibodies (DAKO, #P0447 and #P0448). Proteins were visualised using Prime enhance chemiluminescence (ECL) western blotting substrate (GE Healthcare) and quantified by densitometric analysis using ImageJ software.

**Table 1:** Primary antibodies.

Primary antibodies				
**Antibody**	**Company**	**Catalogue number**	**Source**	**WB dilution**
β-tubulin	Protein Tech	66240-1-1g	Mouse	1:2000
GAPDH	Santa Cruz Biotechnology	MAB374	Mouse	1:2000
Hsp90	Santa Cruz Biotechnology	SC-13119	Rabbit	1:1000
PAK1	Cell Signaling	2602S	Rabbit	1:1000
PAK2	Cell Signaling	2608S	Rabbit	1:1000
PAK4	Cell Signaling	62690S	Rabbit	1:2000
EGFR	Cell Signaling	4267S	Rabbit	1:1000
PD-L1, clone 5H1	Millipore	MAB1115	Mouse	1:1000
PAK3	Cell Signaling	2609S	Rabbit	1:1000
PAK5 (PAK7)	Protein Tech	66961-1-1g	Mouse	1:1000
PAK6	Gene Tex	GTX127915	Rabbit	1:1000
HER2 (29D8)	Cell Signaling	2165	Rabbit	1:1000
HER4 (111B2)	Cell Signaling	4795	Rabbit	1:500
β-Catenin (D10A8) XP®	Cell Signaling	8480S	Rabbit	1:1000
pβ-catenin (S675)	Cell Signaling	9567S	Rabbit	1:1000
Akt (pan) (C67E7)	Cell Signaling	C67E7	Rabbit	1:1000
pAKT (S473)	Cell Signaling	9271S	Rabbit	1:1000
MAPK (ERK1/ERK2)	Cell Signaling	9102S	Rabbit	1:1000
Phospho-p44/42 MAPK (ERK1/ERK2) (Thr202/Tyr204)	Cell Signaling	9106S	Mouse	1:2000

### Isolation and expansion of human T cells

Buffy coats from healthy donors were purchased from the National Blood Transfusion Service. Peripheral blood mononuclear cells (PBMCs) isolated with Ficoll-Paque density separation (Cytiva Life Sciences) were magnetically labelled with human CD8 microbeads (Miltenyi Biotec) and naïve cells separated using positive selection (MACS^®^), with at least 94% positivity for CD8α. A portion was activated for 7–10 days using Dynabeads™ Human T-Activator CD3/CD28 (Thermo Fisher) at a bead to cell ratio of 1:1. Naïve and activated CD8^+^ T cells were cultured in complete medium (RPMI-1640 and 5% human serum), and human IL-2 (100IU/mL) was added for T cell expansion.

### MTT viability assay

Cell viability was assessed by evaluating the [3-(4,5-dimethylthiazol-2-yl)-2,5-diphenyl-tetrazolium bromide] (MTT) reduction to formazan. Cells were seeded in triplicate in 96-well plates at a density of 4 × 10^4^ (Ovsaho) and 3 × 10^4^ (Kuramochi) cells per well. The media was aspirated, and cells were incubated for 3 h at 37 °C with 50 μl of MTT solution (2 mg/ml), protected from light. Formazan crystals were dissolved by adding 50 μl of DMSO to each well, and absorbance was measured at 570 nm.

### Co-culture of ovcan cells and T cells

Ovcan cells were grown in 96-well plates in triplicate with media as a control, seeded at an optimal density predetermined by MTT assay. Once the ovcan cells reached exponential growth, naïve and activated CD8^+^ T cells were added to each of the triplicate wells at varying ratios. The ovcan cells, and naïve and activated CD8^+^ T cells were all also seeded in triplicate wells alone as baseline controls. After 48 h, the cells were processed as previously described in the “MTT viability assay”. The growth rate for the ovcan cells was plotted on a bar graph with each bar representing a different experimental or controlled condition. The effects of PAK inhibition on PBMC CD8-mediated killing of HGSC cells were tested by two methods: concurrent treatment and pre-treatment. During concurrent treatment, Pan PAKi, Group II, and Group I inhibitors (at both 1 µM and 5 µM) were added simultaneously with purified CD8^+^ T cells (naïve or activated, as indicated above) to Ovsaho and Kuramochi cells. In pre-treatment conditions, Ovsaho and Kuramochi cells were treated with each of the three PAK inhibitors (at both 1 µM and 5 µM) for 48 h prior to adding PBMC-derived CD8^+^ T-cells for an additional 48 h. PAK inhibitors and DMSO controls were replaced with complete medium before adding the T cells in all co-culture experiments, and medium was replaced in ovcan control conditions.

### 3D spheroid assay

Tumour cells were counted and seeded in ultra-low adhesion U-Bottom plates (Corning) with three replicates per condition. A 200 µl cell suspension of cells and media was added per well and the plate was centrifuged at RT for 5 m at 500 × *g* and the plate was incubated at 37 °C, 5% CO₂ for 72 h. On Day 3 of spheroid formation, 150 μl of media was removed from each well and 100 μl/well of collagen mixture (1 mg/ml rat tail type I collagen, FBS, 5X DMEM, NaOH, and H_2_O), was added to the plate on ice. The plate was then left for 60–90 m at RT to allow collagen polymerisation. Complete media or inhibitors (at 10 µM) were added at 50 μl/well and the plate was kept at 37 °C, 5% CO₂ for the remainder of the experiment. Invading cells were counted manually in ImageJ.

### Transwell migration assay

Cells were starved by culturing in serum-free medium for 6 h prior to assay. 600 μl of either complete medium, DMSO-treated medium, Pan PAKi (5 µM) medium, or serum-free medium (negative control) was added to a 24-well plate, and 8 μm transwell inserts (Corning; ref. 3422) were placed at the top of each well. Kuramochi cells were seeded at 4 × 10^4^ per insert in 200 μl serum-free medium, and the plate was incubated at 37 °C, 5% CO₂ for 24 h. The insert was then carefully removed and washed 2X with 500 μl 1XPBS. Cells were fixed with 500 μl 4%PFA for 30 m at RT. Non-migrated cells were removed with a cotton swab, and the insert was washed 3X with 500 μl 1XPBS. Cells were then stained with 500 μl of 0.1% crystal violet for 30 m at RT. For quantification, 33% acetic acid was added to each well and incubated for 30 m at RT. The solution was then transferred to a 96-well plate, and absorbance was measured at 579 nm.

### Flow cytometry

Flow cytometry experiments were performed after staining of samples with antibodies specific to human CD8α, CD274 (PD-L1), and CD279 (PD-1) (Biolegend) conjugated with FITC, APC, and BV421 fluoro-chromes, respectively. Viability was assessed with eFluor™ 780 (eBioscience^™^). Samples were acquired with FACSFortessa (BD Biosciences) and CytoFlex (Beckman) cytometers using BD FACSDiva and CytExpert software, respectively. For each sample, a minimum of 5000 events were acquired, and data were analysed using FlowJo v10.8.1 and GraphPad Prism 9.2.0 (283).

### Supernatant analysis with human magnetic Luminex assay

All reagents were prepared according to the instructions manual of a custom kit (R&D Systems). Supernatant collected from cytotoxicity assays were retrieved from −80 °C and thawed on ice before centrifuging 4 m at 16,000 rcf at RT. The supernatant was collected and diluted 1:2 with kit diluent and plated at 50μl/well in duplicate, along with standard curve samples. A total of 50 µl of diluted microparticle cocktail was added to each well, and samples were incubated for 2 h in the dark at RT at 800 rpm. Samples were washed 3 times and 50 µl of diluted Biotin-antibody cocktail was added to each well. Samples were then incubated for 1 h in the dark at RT at 800 rpm. After, the plate was washed 3 times and 50 µl of diluted Streptavidin-PE was added to each well. Samples were again incubated for 30 min in the dark at RT on a shaker at 800 rpm. The plate was washed 3 times, and 100 µl of wash buffer was added to each well. Then the plate was wrapped in foil and incubated for 2 m at RT on a shaker at 800 rpm. The plate was read within 90 m using a Luminex 3D, and the microparticles were resuspended immediately prior to reading.

### Human granzyme B ELISA

All reagents were prepared according to the instruction manual (U Cy-tech; Cat. No. CT211A). Activated CD8^+^ T cells were incubated with complete medium, DMSO, or Group I PAKi (5 µM), and supernatant samples were diluted 1:4 after collection for plating.

### Statistical methods

Statistical analyses were conducted with GraphPad Prism 10.0.2 (171). For multiple comparisons, one-way ANOVA was used. Alternatively, Student’s *t* tests were used to calculate statistical significance. Post hoc analyses were also used, where appropriate. All graphs show mean values and standard deviation (SD) or median values and ranges. The values were considered statistically significant if *p* value < 0.05.

### Ethics approval and consent to participate

Human blood was obtained from the National Blood Transfusion Service UK under a Human Tissue Authority licence to CMW. Blood donation and its use in research are governed by NHS Blood and Transplant (NHSBT) under the Human Tissue Act 2004 and the Blood Safety and Quality Regulations (BSQR 2005). Donors give informed consent at the time of donation, which covers both transfusion and potential anonymised use in research. Ethical oversight is provided by NHSBT and the SaBTO (Safety of Blood, Tissues and Organs) committee, ensuring compliance with national law, EU directives, and research ethics frameworks.

## Results

### HGSC cells express PAK family kinases and surface PD-L1

**Fig. 1:**
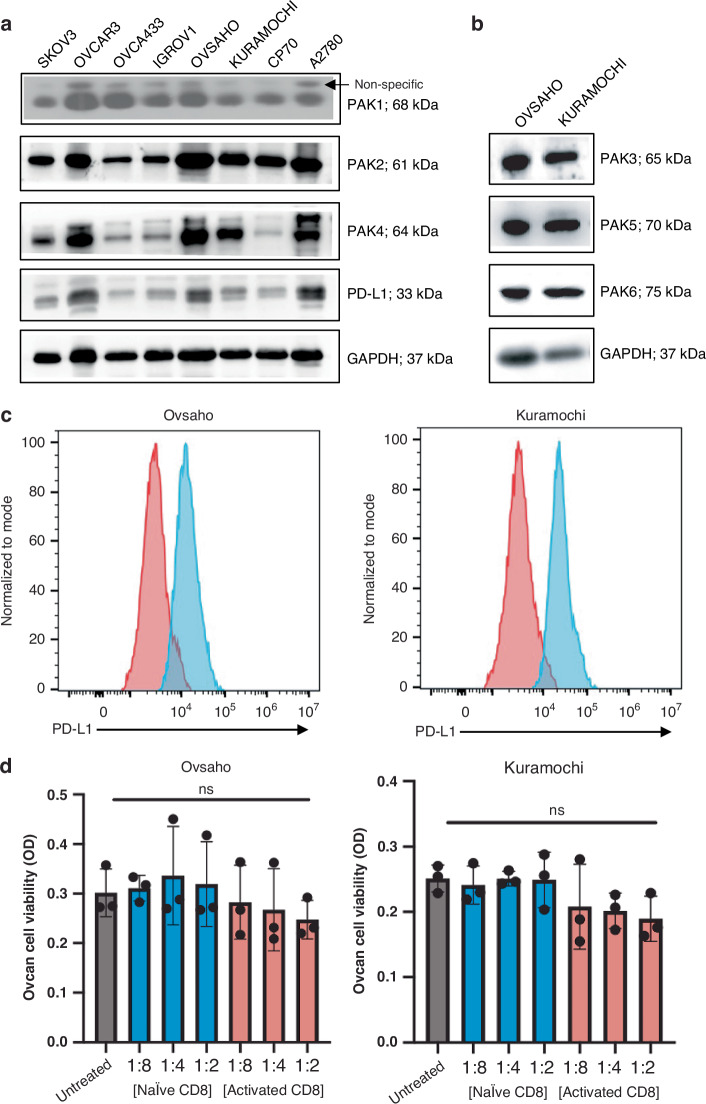
HGSC cells are viable targets of PAKi and αPD-(L)1 combination therapy. **a** Western blot showing PAK1, PAK2, PAK4, and PD-L1 expression across ovcan cell panel. GAPDH was used as a loading control. **b** Western blot showing PAK3, PAK5, and PAK6 expression levels in Ovsaho and Kuramochi HGSC cell lines. GAPDH was used as a loading control. **c** Cytometry plots show an increase in surface PD-L1 in stained populations (blue peaks) compared to isotype control populations (red peaks) in Ovsaho, *left plot*, and Kuramochi cells, *right plot*. **d** Serial cytotoxicity assay showing viability of Ovsaho (left) and Kuramochi cells (right) after co-culture with naïve and activated CD8^+^ T cells at varying T:E (Tumour:Effector cell) ratios for 48 h. Data are presented as mean ± SD. Statistical significance was calculated with one-way ANOVA: ns, not significant, *p*  >  0.05; *p  <  0.05; **p  <  0.01; ***p  <  0.001. All figures represent *N* = 3 biologically independent experiments.

Although several studies have identified commercial human cell lines most representative of HGSC, previous studies on the effects of PAK inhibitors in ovcan did not utilise cell lines most representative of the HGSC subtype [3, 9, 15]. Key components of HGSC elucidated by genomic profiling are a good correlation between the copy-number profile of the cell line and the mean copy-number profile of HGSC tumour samples, a low frequency of non-synonymous mutations in protein-coding genes, the presence of a *TP53* mutation, and the absence of mutations in seven ‘non-HGSC’ genes altered in other ovcan subtypes [3]. Ovsaho and Kuramochi cells are commonly accepted as highly representative of HGSC [27]. based on these criteria. However, these ovcan lines have not been screened comparatively for PAK family and PD-L1 expression. We found that HGSC representative cell lines Ovsaho and Kuramochi have relatively high expression of these PAKs and total PD-L1 compared to other ovcan lines (Fig. 1a). Ovsaho and Kuramochi cells also express PAK3, PAK5, and PAK6 (Fig. 1b), and surface expression of PD-L1 was confirmed in both cell lines by FACS analysis (Fig. 1c), as well as surface expression of PD-L2 (Supplementary Fig. 1). Low-level (non-significant) tumour cell killing was observed when pre-activated, but not naïve, CD8^+^ T-cells were co-cultured with Ovsaho and Kuramochi ovcan cells (Fig. 1d). This is likely due to alloreactivity of these CD8^+^ T-cells since it was accompanied by degranulation (Granzyme B release) and secretion of pro-inflammatory cytokines (Supplementary Figs. 2 and 3).

### PAKi disrupts downstream signalling in HGSC cells

**Fig. 2:**
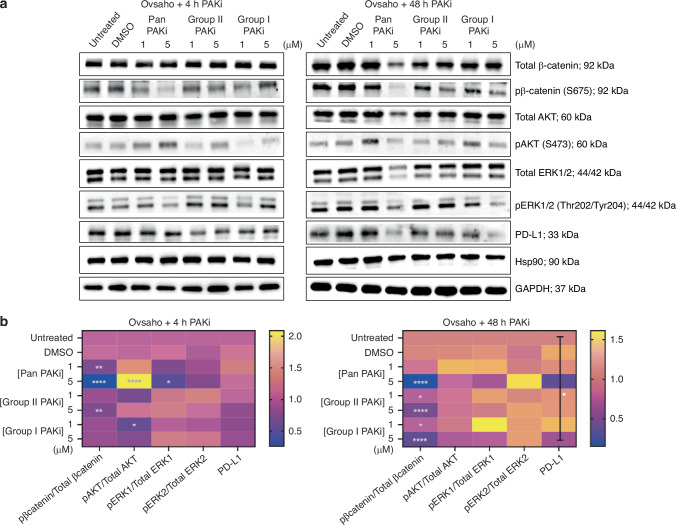

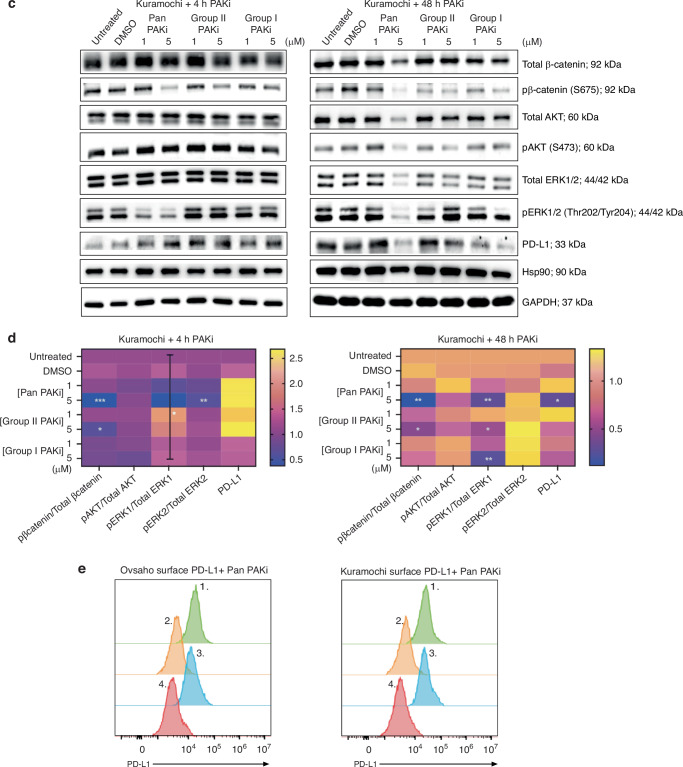
Impact of PAKi on downstream molecular pathways and surface PD-L1 in HGSC. **a** Western blot analysis of indicated proteins in Ovsaho cells treated with the indicated dose of Pan PAKi, Group II PAKi, and Group I PAKi for 4 h (left) or 48 h (right). Hsp90 and GAPDH were used as loading controls. **b** Heat map summarising the effects of PAKi on downstream pathways in Ovsaho cells at 4 h (upper), and 48 h (lower). Statistical significance was calculated with a two-way ANOVA; *p < 0.05. At 4 h, row factor (treatment) was significant at *p* = 0.0016, and the interaction between row and column factors was significant at *p* < 0.0001; at 48 h, row factor (treatment) and column factor (protein of interest) were both significant at *p* < 0.0001, and the interaction between row and column factors was significant at *p* = 0.0051. For each protein of interest, statistical significance was calculated with one-way ANOVA, and post hoc analyses: blank, not significant; *p  <  0.05; **p  <  0.01; ***p  <  0.001. **c** Western blot analysis of indicated proteins in Kuramochi cells treated with the indicated dose of Pan PAKi, Group II PAKi, and Group I PAKi for 4 h (left) or 48 h (right). Hsp90 and GAPDH were used as loading controls. **d** Heat map summarising the effects of PAKi on downstream pathways in Kuramochi cells at 4 h (upper), and 48 h (lower). Statistical significance was calculated with a two-way ANOVA; *p < 0.05. At 4 h, column factor (protein of interest) was significant at *p* < 0.0001, and the interaction between row and column factors was significant at *p* = 0.0127; at 48 h, row factor (treatment) was significant at *p* < 0.0001, and column factor (protein of interest) was significant at *p* = 0.0002. For each protein of interest, statistical significance was calculated with one-way ANOVA, and post hoc analyses: blank, not significant; *p  <  0.05; **p  <  0.01; ***p  <  0.001. **e** Histogram overlays show surface PD-L1 expression of Ovsaho (left) and (right) Kuramochi cells after 48 h Pan PAKi treatment. Numbered peaks represent (1) HGSC + 5 µM Pan PAKi, (2) HGSC + 5 µM Pan PAKi isotype control, (3) HGSC control (untreated cells), and (4) HGSC isotype control (untreated cells) conditions. All figures represent *N* = 3 biologically independent experiments.

Having established that Ovsaho and Kuramochi cells express PAK family proteins at detectable levels, we subsequently evaluated the biochemical response to PAK inhibition. This study is the first to explore the effects of three group-specific PAK inhibitors targeting Group I, Group II, and Pan-PAK kinases in HGSC cell lines. PAK1 is known to promote transcriptional activity of β-catenin, and PAK4 transports it between the nucleus and cytoplasm [28], and active β-catenin also plays a role in ovcan angiogenesis, immune evasion [29], and ovcan EMT regulation via PAK1 [30]. AKT activity regulates cell survival and growth in ovcan [31], and PAK1 and PAK2 are necessary for phosphorylation of AKT in several other cancer types [32]. PAKs also have a well-characterised role in ERK activation [33, 34]; indeed, PAK1 kinase inhibition has been shown to significantly decrease downstream ERK phosphorylation activity in OVCAR3 and OV90 [9], and ERK signalling can contribute to ovcan progression [35, 36], promotion of cell survival and proliferation, and contribution to anchorage-independent growth in ovcan through E-cadherin [37]. In our study, a higher concentration of PAKi decreased pERK1 activity after 48 h in Ovsaho (Fig. 2a, b) and Kuramochi cells (Fig. 2c, d); comparatively, pERK2 activity remains more stable. Intriguingly, there is a significant decrease in pβ-catenin activity after 4 h that persists after 48 h PAKi in both Ovsaho and Kuramochi cells. There is also a distinct increase in AKT activity after 48 h with lower concentrations of PAKi, while a decrease in AKT activity is seen with higher concentrations of PAKi. However, there is no significant decrease in ovcan cell viability after PAKi, although there is a trend towards reduced viability after Pan PAKi (5 μM) (Supplementary Fig. 4A, B); and viability is maintained for CD8^+^ T cells treated with PAKi (Supplementary Fig. 5).

### Surface levels of PD-L1 are modulated in PAKi treated cells

Pan and Group 1 PAKi treatment (5 µM) lowered total PD-L1 expression at 48 h in both Ovsaho (Fig. 2a, b) and Kuramochi cells (Fig. 2c, d). Whilst total PD-L1 levels were decreased, surface expression of PD-L1 was unexpectedly maintained in the presence of PAKi for both HGSC lines at this timepoint (Fig. 2e and Supplementary Fig. 6A, B). Further examination showed surface PD-L1 increased on HGSC cells with PAKi from 4 to 48 h (Supplementary Fig. 7).

### PAKi decreases 3D HGSC cell invasion

**Fig. 3:**
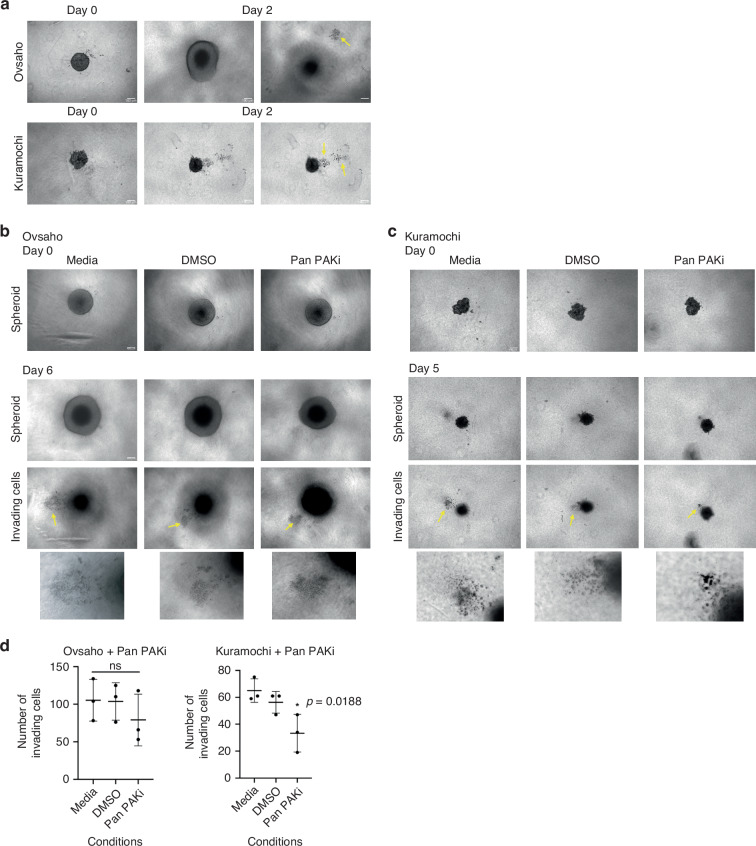
PAKi decreases HGSC cell invasion and increases surface trafficking of PD-L1. **a** 3D spheroid invasion in HGSC cells. Ovsaho cells, upper panel, and Kuramochi cells, lower panel, were seeded in ultra-low adhesion plates and formed spheroids in collagen. Day 2 images show HGSC spheroid (middle images) and collective migration of HGSC cells away from spheroid core (right-side images; indicated by arrows). Scale bar is 100 μm. **b** Ovsaho cell invasion before (Day 0) and after (Day 6) 10 µM Pan PAKi treatment. Day 6 images show Ovsaho spheroids with medium and DMSO controls (upper panel), and collective migration of Ovsaho cells away from the spheroid core (lower panel), indicated by arrows, with zoomed images below. Scale bars are 100 μm. **c** Kuramochi cell invasion before (Day 0) and after (Day 5) 10 µM Pan PAKi treatment. Day 5 images show Kuramochi spheroids with medium and DMSO controls (upper panel), and collective migration of Kuramochi cells away from the spheroid core (lower panel), indicated by arrows, with zoomed images below. Scale bars are 100 μm. **d** Quantification of HGSC 3D cell invasion in Ovsaho, left, and Kuramochi cells, right, after 10 µM Pan PAKi treatment. Data are presented as mean ± SD with each point corresponding to a single experiment. Statistical significance was calculated with one-way ANOVA and post hoc analyses: ns, not significant, *p*  >  0.05; *p  <  0.05; **p  <  0.01; ***p  <  0.001. All figures represent *N* = 3 biologically independent experiments.

To fully understand the cellular response to PAK inhibition and given that PAK inhibition did not reduce the viability of Ovsaho and Kuramochi cells, we further tested whether PAK inhibition might impact invasive behaviour. To test this, a 3D spheroid assay was used to assess invasive potential by allowing multi-directional cellular invasion into surrounding collagen. This model was favoured as the invasive properties of the spheroids recapitulate the recognised primary method of local invasion and metastasis [27, 38]. This spheroid assay is novel in examining the 3D effects of PAKi on HGSC cells in collagen, where other studies have investigated the effect of other chemotherapeutics like cisplatin in other matrices like Matrigel [39, 40]. Ovsaho cells formed compact spheroids and notably, 48 h after adding collagen [Day 2 of invasion] there was clear evidence of invasion from the spheroid, with cells moving collectively up towards the surface (Fig. 3a, upper panel). Contrastingly, Kuramochi spheroids were more loosely formed, where individual cells could still be distinguished at the spheroid edge, and cell invasion was also noticeable after 48 h in collagen, with two distinct groups of cells invading upwards from the spheroid body (Fig. 3a, lower panel). Ovcan invasion usually occurs by passive dissemination, where shed cells from the primary tumour site form spheroids within the peritoneal cavity [38, 41]. These cells can then individually or collectively migrate through the mesothelial lining of the abdominal cavity, seeding a secondary site [42, 43]. Ovsaho and Kuramochi cells both rapidly formed spheroids, demonstrating continued invasion by Day 6 (Fig. 3b) or Day 5 (Fig. 3c), respectively. As previously shown, invasion in both cell lines appears to occur by collective migration upwards from the spheroids. Pan PAKi treatment significantly decreased cellular invasion in Kuramochi cells, while a similar non-significant trend was observed with Ovsaho cells (Fig. 3d). This effect was further confirmed in a transwell migration assay (Supplementary Fig. 8).

Having established that Ovsaho and Kuramochi cells express PAK family proteins and respond to PAK inhibition, we then sought to understand whether PAKi could improve the CD8-mediated killing of ovcan cells from baseline levels.

### PAKi limits HGSC immune escape

**Fig. 4:**
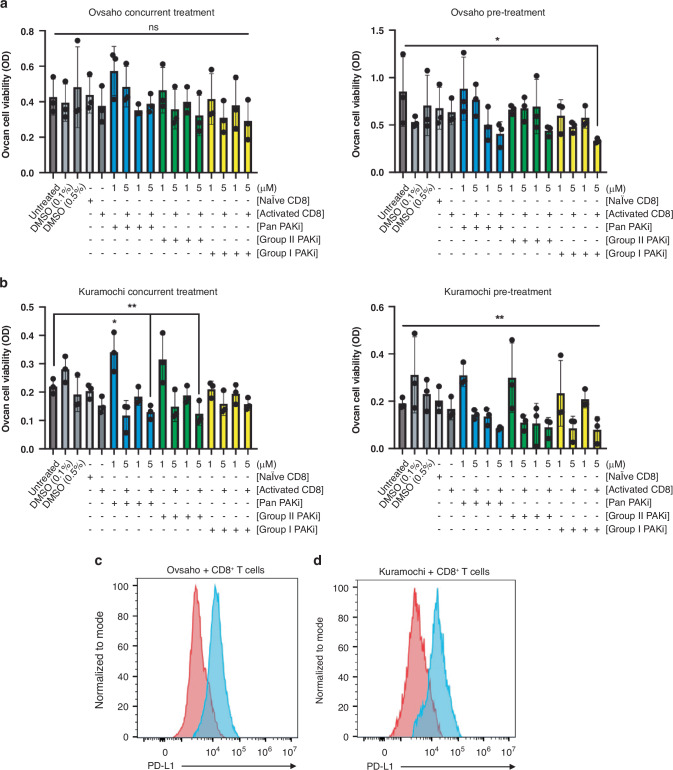
PAKi treatment sensitises HGSC cells to activated CD8^+^ T cell killing. Plots show MTT viability results of (**a**) Ovsaho and (**b**) Kuramochi cells after PAKi concurrent treatment and PAKi pre-treatment followed by co-culture with PBMC isolated CD8^+^ T cells at 1:4 (T:E). Statistical significance was calculated with one-way ANOVA and post hoc analyses comparing untreated cells with other experimental conditions unless otherwise indicated: ns, not significant, *p*  >  0.05; *p  <  0.05; **p  <  0.01; ***p  <  0.001. Plots show PD-L1 surface expression on (**c**) Ovsaho and (**d**) Kuramochi cells after 48 h co-culture with activated CD8^+^ T cells, with an increase in surface PD-L1 in stained populations (blue peaks) compared to isotype control populations (red peaks). All figures represent *N* = 3 biologically independent experiments.

In addition to limiting invasion, PAK4 inhibition has been shown to increase T cell infiltration and decrease tumour burden in melanoma [44]. The effects of PAKi on T cell-mediated killing have not been studied in ovcan. Therefore, we developed a new platform to evaluate the effects of PAKi on CD8^+^ T cell cytotoxicity by two methods: concurrent treatment and pre-treatment. In the pre-treatment setting, Ovsaho and Kuramochi cells were treated with PAKi for 48 h prior to adding PBMC-derived CD8^+^ T cells, as HGSC cellular invasion was significantly reduced at this timepoint. We found concurrent treatment only slightly improved CD8-mediated killing of Ovsaho cells, while pre-treatment led to a significant decrease in Ovsaho viability compared to PAKi alone (Fig. 4a). Notably, both concurrent and pre-treatment significantly improved CD8^+^ T cell-mediated killing of Kuramochi cells (Fig. 4b), although overall percent killing was higher after pre-treatment (Supplementary Tables 1 and 2). Simultaneously, surface PD-L1 is maintained on Ovsaho (Fig. 4c) and Kuramochi (Fig. 4d) cells after co-culture with CD8^+^ T cells.

### PAKi and αPD-1 combination therapy improves CD8^+^ T cell killing of HGSC cells

**Fig. 5:**
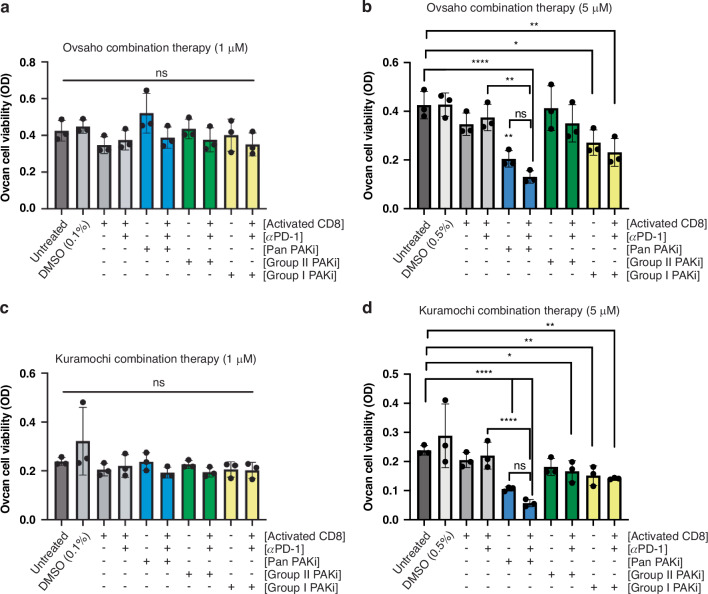
PAKi and αPD-1 combination therapy improves CD8^+^ T cell killing of HGSC cells. **a** Plot shows MTT viability results from the combination therapy of 1 µM PAKi and PBMC CD8s (1:4, T:E) + αPD-1 (500 ng) in Ovsaho cells treated with indicated Pan PAKi, Group II PAKi, or Group I PAKi. **b** Plot shows MTT viability results from combination therapy of 5 µM PAKi and PBMC CD8s (1:4, T:E) + αPD-1 (500 ng) in Ovsaho cells treated with indicated Pan PAKi, Group II PAKi, or Group I PAKi. **c** Plot shows MTT viability results from combination therapy of 1 µM PAKi and PBMC CD8s (1:4, T:E) + αPD-1 (500 ng) in Kuramochi cells treated with indicated Pan PAKi, Group II PAKi, or Group I PAKi. **d** Plot shows MTT viability results from combination therapy of 5 µM PAKi and PBMC CD8s (1:4, T:E) + αPD-1 (500 ng) in Kuramochi cells treated with the indicated Pan PAKi, Group II PAKi, or Group I PAKi. Ovsaho and Kuramochi cells were pre-treated for 48 h with PAK inhibitors before adding activated PBMC-derived CD8^+^ T cells and 500 ng pembrolizumab for another 48 h. Data are presented as mean ± SD. Statistical significance was calculated with one-way ANOVA and post hoc analyses comparing untreated cells with other experimental conditions unless otherwise indicated: ns, not significant, *p*  >  0.05; *p  <  0.05; **p  <  0.01; ***p  <  0.001. All figures represent *N* = 3 biologically independent experiments.

Our findings suggest that PAKi can improve CD8 T cell-mediated killing, but PD-L1 still serves as an inhibitory break on this activity. We hypothesised that a combination of PAKi and PD-1 blockade could further bolster cytotoxic CD8^+^ T cell killing of HGSC cells. Combination therapy applying kinase inhibition and PD-1 blockade has been proposed as a potential therapeutic strategy in advanced melanoma, colon, and renal cell cancers, as it could aid the immune system in targeting tumours [44, 45]. PAKs have also been previously characterised for their role in immune cell infiltration in several cancer types, with an emphasis on inhibition alone or in combination with an immunotherapeutic [46–50]. In our study, we utilised a novel combination of PAKi pre-treatment and PD-1 checkpoint blockade with pembrolizumab [21]. Our results show that low-dose PAKi (1 µM) combination therapy did not significantly impact cell viability, even in the presence of activated CD8^+^ T cell killing of Ovsaho (Fig. 5a) and Kuramochi (Fig. 5c) cells. In comparison, a higher dose (5 µM) of Pan PAKi in combination therapy significantly augmented HGSC cell killing (Fig. 5b, d). CD8^+^ T cell killing was remarkably enhanced with Pan PAKi combination therapy and increased from 9 to 69% in Ovsaho co-cultures and from 19 to 76% in Kuramochi co-cultures (Supplementary Table 3).

## Discussion

**Fig. 6:**
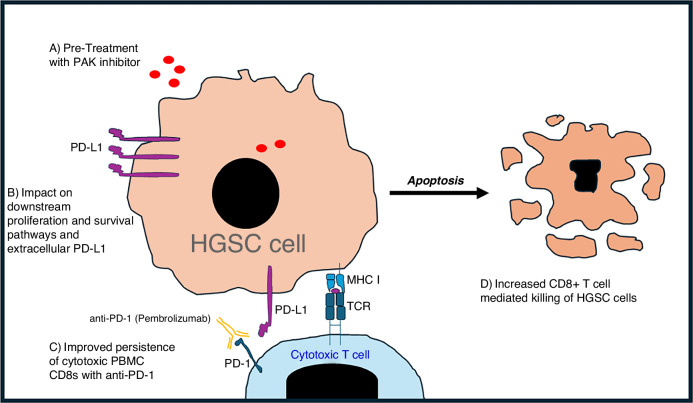
Schematic of PAKi and αPD-1 combination therapy in HGSC. PAKi pre-treatment sensitises HGSC cells, and downstream phosphorylation pathways like pβ-catenin, pAKT, and pERK1/pERK2 are impacted. As a protective mechanism, extracellular PD-L1 is maintained in HGSC cells, and when αPD-1 is added to T cells, CD8^+^ T cell-mediated killing is enhanced. This model proposes that a combination of PAKi and αPD-1 improves HGSC cell killing and limits dissemination better than either monotherapy alone.

We have demonstrated here that PAKi pre-treatment can sensitise HGSC cells to T cell-mediated killing. We have evidence that PAKi can disrupt downstream signalling, while surface PD-L1 is still maintained as an inhibitory mechanism. CD8^+^ T cell-mediated killing is further enhanced when αPD-1 is added (Fig. 6).

This study is the first to show the effects of combined PAKi, activated CD8^+^ T cells and αPD-1 therapy in HGSC cell models. PAK and PD-L1 expression profiles across ovarian cancer cell lines were characterised here for the first time, as well as PD-L1 surface expression and the invasive ability of HGSC representative lines. A pre-clinical co-culture cytotoxicity assay was developed to assess the suitability of these HGSC cell lines as models to test combined PAKi and PD1 receptor blockade in HGSC, and to test immune escape of HGSC cells from cytotoxic CD8^+^ T cell-mediated killing.

PAK family kinases are known regulators of cancer metastasis and invasion, and expression of PAK1, PAK2, and PAK4 across the cell panel is consistent with studies in the literature reporting overexpression of these PAKs in ovcan [6, 10, 51]. Ovsaho cells have higher relative expression of PAK1 than Kuramochi cells, and both have high relative PAK4 expression. The prolonged effect of PAKi on decreasing downstream β-catenin and ERK1 phosphorylation activity after 48 h is notable, but not improbable, given the 2016 study by Prudnikova et al., where Group I PAKi (FRAX1036) significantly reduced pERK1/2 activity in OVCAR3 and OV90 cells after 24 h treatment [9]. The significant decrease in levels of β-catenin phosphorylation in both Ovsaho and Kuramochi cells after 4 and 48 h exposure to PAKi is novel. This suggests that PAK1 and PAK4 are indeed involved in its transcriptional activity and mediate its phosphorylation at S675 in HGSC in addition to other cancer types [52, 53]. These results also suggest that β-catenin activity in HGSC cells is in part dependent upon PAK1 and/or PAK4. The effects of PAKi on other known downstream pathways, like PI3K/AKT and Wnt/β-catenin, have also been reported as mechanisms of HGSC cell survival and metastasis [29, 31]. Impacts on β-catenin activity are not unexpected, as PAK1 is already known to promote transcriptional activity of β-catenin and PAK4 shuttles the protein between the nucleus and cytoplasm [28], contributing to ovcan angiogenesis and immune evasion [29].

The minimal effect of all three PAK inhibitors on AKT phosphorylation in both lines is surprising, given other studies have reported PAK1 and PAK4-mediated phosphorylation of AKT at S473 in other cancer types [54, 55]. AKT activity can regulate cell survival and growth in ovcan [31], and PAK1 and PAK2 are necessary for phosphorylation of AKT in several other cancer types [32]. The lack of response could be due to PAK1, or even PAK4-mediated activation of AKT in a kinase-independent manner [56, 57], which has not yet been reported in ovcan, or the upregulation of compensatory pathways in these cells. Differences in pERK1/2 activity between Ovsaho and Kuramochi cells after PAKi are partially unexpected, because while PAK1 has a well-characterised role in MEK/ERK activation [58], other studies have reported inconsistent reduction in ERK activation in malignant peripheral nerve sheath tumours [59], and other ovcan lines [9]. The decrease in ERK1 activity in Kuramochi cells is more pronounced than in Ovsaho cells after 48 h PAKi, and this could be a result of PAK1 phosphorylating ERK through kinase-independent activity in Ovsaho cells [60], a finding that has not yet been reported in ovcan. In addition to PAK1, PAK4 can also hinder apoptosis in cancer cells, acting via both kinase-independent and kinase-dependent manners through activation of TNF-α and ERK pathways [61]. ERK is especially critical for ovcan progression [36], and AKT promotes ovcan survival [31]. Consequently, these results may indicate a possible role for PAKs in primarily driving HGSC proliferation over survival.

Previous studies have examined the effects of PARPi on 3D HGSC invasion with agarose plates, and the effects of dual inhibition of PAK4 and NAMPT on non-HGSC 3D spheroids [62, 63], but this study is the first to explore 3D HGSC invasion into type I collagen, which is preferred over other ECM proteins for adhesion and invasion of ovcan spheroids [64, 65]. In the 3D invasion assay, Ovsaho and Kuramochi spheroids formed quite readily and were compact, possibly from a preference towards colony formation. Spheroid formation in HGSC cells is well-established as a precursor to ovcan invasion [66], although this study is the first to examine 3D invasion of HGSC spheroids fully immersed in collagen. Remarkably, cell invasion was noticeable as early as 48 h after adding collagen, and the Ovsaho and Kuramochi cells appeared to collectively migrate in a single direction. This is an important observation, given that in HGSC peritoneal dissemination, a cluster of ovcan cells detach, circulate through the peritoneal fluid and collectively migrate through the mesothelial layer of the abdominal cavity [67]. Invasion is a prominent characteristic of the HGSC subtype [68], and the significant decrease in Kuramochi 3D cell invasion after Pan PAKi is notable and highlights a possible role for PAKs in HGSC dissemination for the first time. PAK expression could be a predictor of PAKi success in limiting 3D ovcan invasion, where the level of Group I or Group II PAK expression could determine the efficacy of Group I, Group II, or Pan PAK inhibitors. As a result, the relatively high PAK1 or PAK4 expression in Ovsaho cells compared to Kuramochi cells could be driving invasion in these cells even after Pan PAKi treatment. Additionally, PAK1 activity is necessary for modulating cell adhesion by E-cadherin [69], which can mediate ERK activity by regulating ovcan proliferation [37]. Consequently, it is possible that higher PAK1 expression in Ovsaho cells facilitates continued invasion through this pathway. Ovsaho cell cultures consistently have higher numbers of invading cells compared to Kuramochi cells, but it is possible that a higher concentration of Pan PAKi could significantly decrease Ovsaho cell invasion.

PAK inhibition has been linked to increased anti-tumour activity in cytotoxic T cells, where PD-L1 expression was downregulated in pancreatic ductal adenocarcinoma (PDA) cells treated with PAKi targeting PAKs 1, 4, 5, and 6 [14]. In the same study, PAKi pre-treatment also sensitised the PDA cells to killing by cytotoxic T cells in co-culture assays. In our study, the significant decrease in total PD-L1 expression after Pan PAKi of Kuramochi cells suggests a novel link between PAKs and the PD-L1 pathway is present in HGSC, and evidence of this is further substantiated by the increase of surface PD-L1 after Pan PAKi in these cells. These results were unexpected because total levels decreased; however, PAKi could be involved in trafficking intracellular PD-L1 to the cell surface, possibly through ERK, AKT, or β-catenin phosphorylation activity [70], at which point PD-L1 could be released from the HGSC cell by secreted exosomes [71, 72]. Several studies have investigated the biogenesis of exosomal PD-L1 and the corresponding impact on immunosuppression and immunotherapy outcomes [73, 74]. Additionally, this effect in Kuramochi cells and not in Ovsaho cells could be due to *KRAS*-amplification in Kuramochi cells [75], as previous studies show *KRAS* downregulates intracellular PD-L1 when pERK activity is inhibited [70], and after PAK inhibition [76].

We found that baseline killing of Ovsaho and Kuramochi cells by activated CD8^+^ T cells can be significantly improved after PAKi pre-treatment and PD-1 blockade. These results suggest Pan PAKi could sensitise HGSC cells intracellularly by targeting and downregulating phosphorylation activity across cell survival pathways. Though surface PD-L1 is maintained, the addition of αPD-1 disrupts this inhibitory brake, further potentiating CD8^+^ T cell killing of HGSC cells. The results support consistent recommendations to combine PAKi with PD-(L)1 immunotherapy [14, 44, 77–79], and in a clinical setting, HGSC patients could receive Pan PAKi pre-treatment orally [80], and after 48 h patients could receive intravenous pembrolizumab.

## Conclusions

In conclusion, this study demonstrates for the first time that the combination of PAK inhibition and PD-1 checkpoint blockade significantly sensitises HGSC cells to activated cytotoxic CD8^+^ T cell-mediated killing. This work supports ongoing recommendations to progress from a mono-therapeutic approach for HGSC treatment to a combination therapy instead, as this could better target HGSC cell invasion and immune escape.

## Supplementary information

SFigures

## Data Availability

The datasets generated during and/or analysed during the current study are available from the corresponding author on reasonable request.
