# Supplementary material for: Investigating PAK inhibition in combination with PD-1 blockade to enhance cytotoxic CD8+ T cell-mediated killing and suppress invasion of ovarian cancer cells
Source: Br J Cancer. 2026 Mar 6;134(9):1248–60. doi: 10.1038/s41416-026-03342-z (PMC13079780; doi:10.1038/s41416-026-03342-z)
Supplement: Supplementary file 1 — SFigures [file 41416_2026_3342_MOESM1_ESM.pdf]

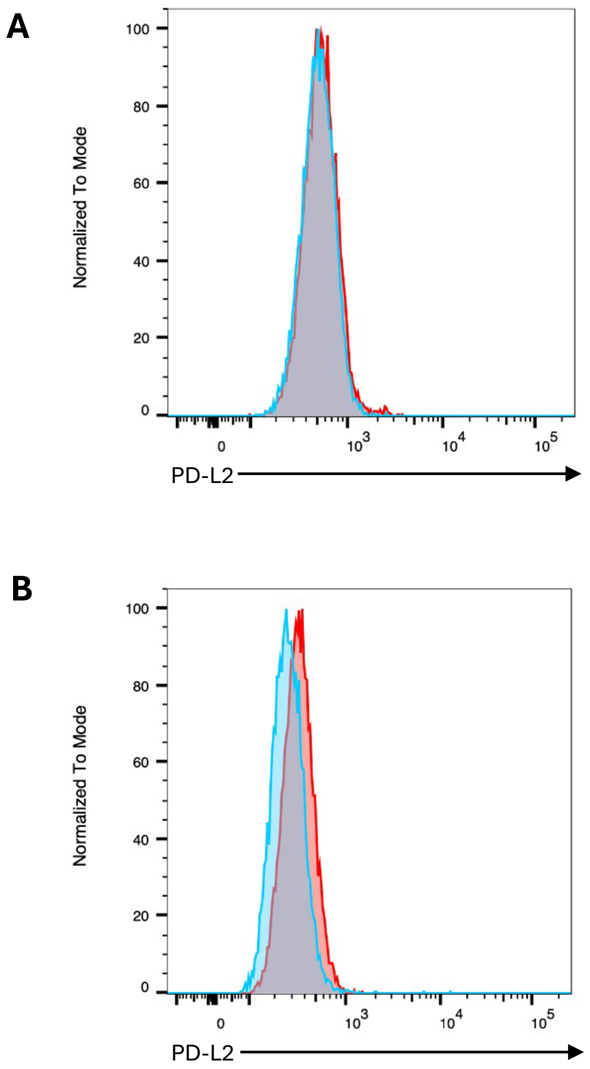

Supplementary Figure 1. Surface level expression of PD-L2 in HGSC cells. Cytometry plots show no difference between stained populations (blue peaks) compared to isotype control populations (red peaks) in **(A)** Ovsaho and **(B)** Kuramochi cells.

A

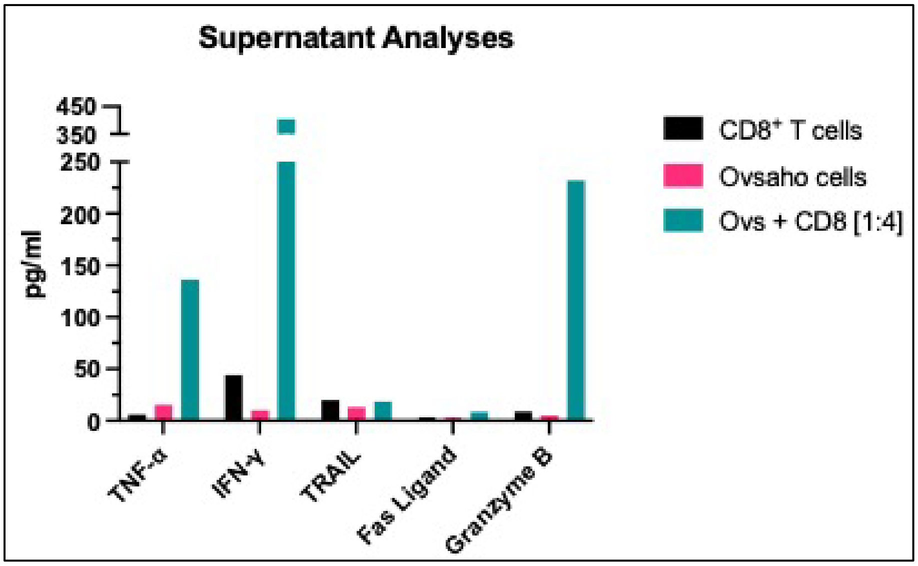

B

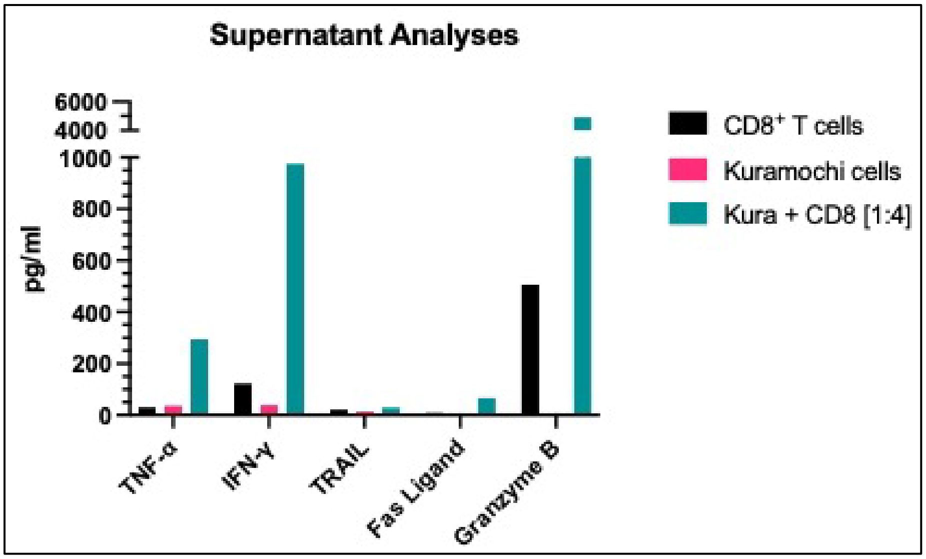

Supplementary Figure 2. Supernatant analyses of Ovcan co-cultures with cytotoxic CD8$^+$ T cells. Cytokines of interest are displayed for (A) Ovsaho and (B) Kuramochi 48hr control and co-culture conditions [Tumour:Effector]. Data shown are mean values and represent at least two samples per control condition and three samples per co-culture condition.

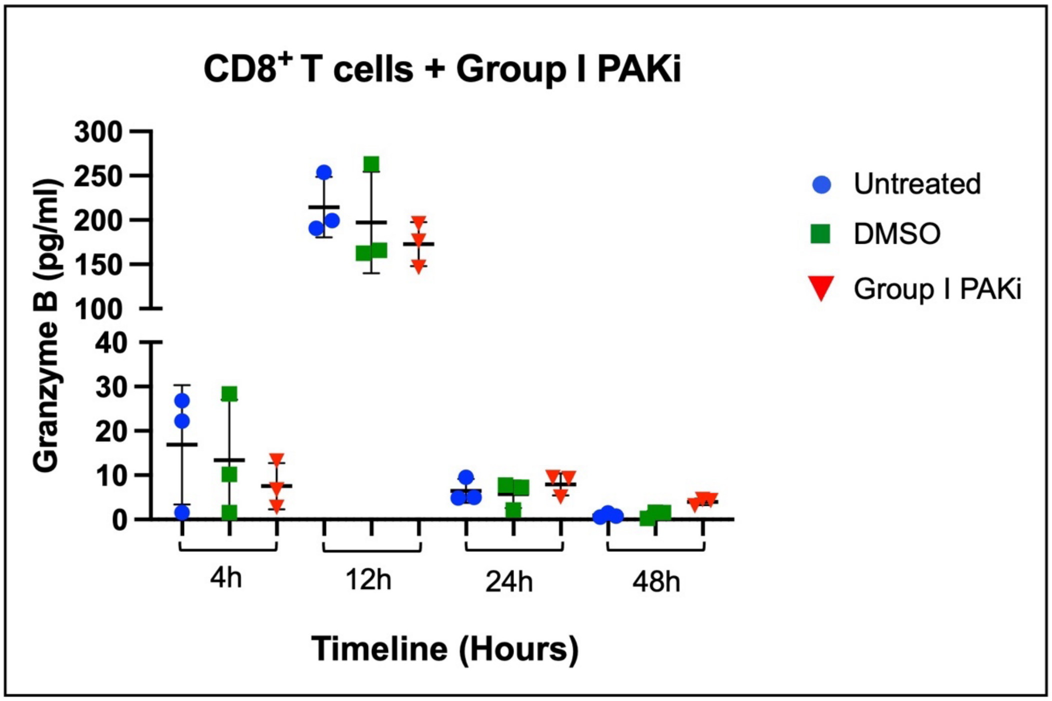

Supplementary Figure 3. Effect of Group I PAKi on CD8$^+$ T cell Granzyme B activity. Plot shows Granzyme B levels in CD8$^+$ T cell supernatant after Group I PAKi (5µM) time course treatment. Data are presented as mean ± SD with untreated (*blue circles*), DMSO (*green squares*), or Group I PAKi (*red triangles*) results. Data represent N=3 biologically independent experiments.

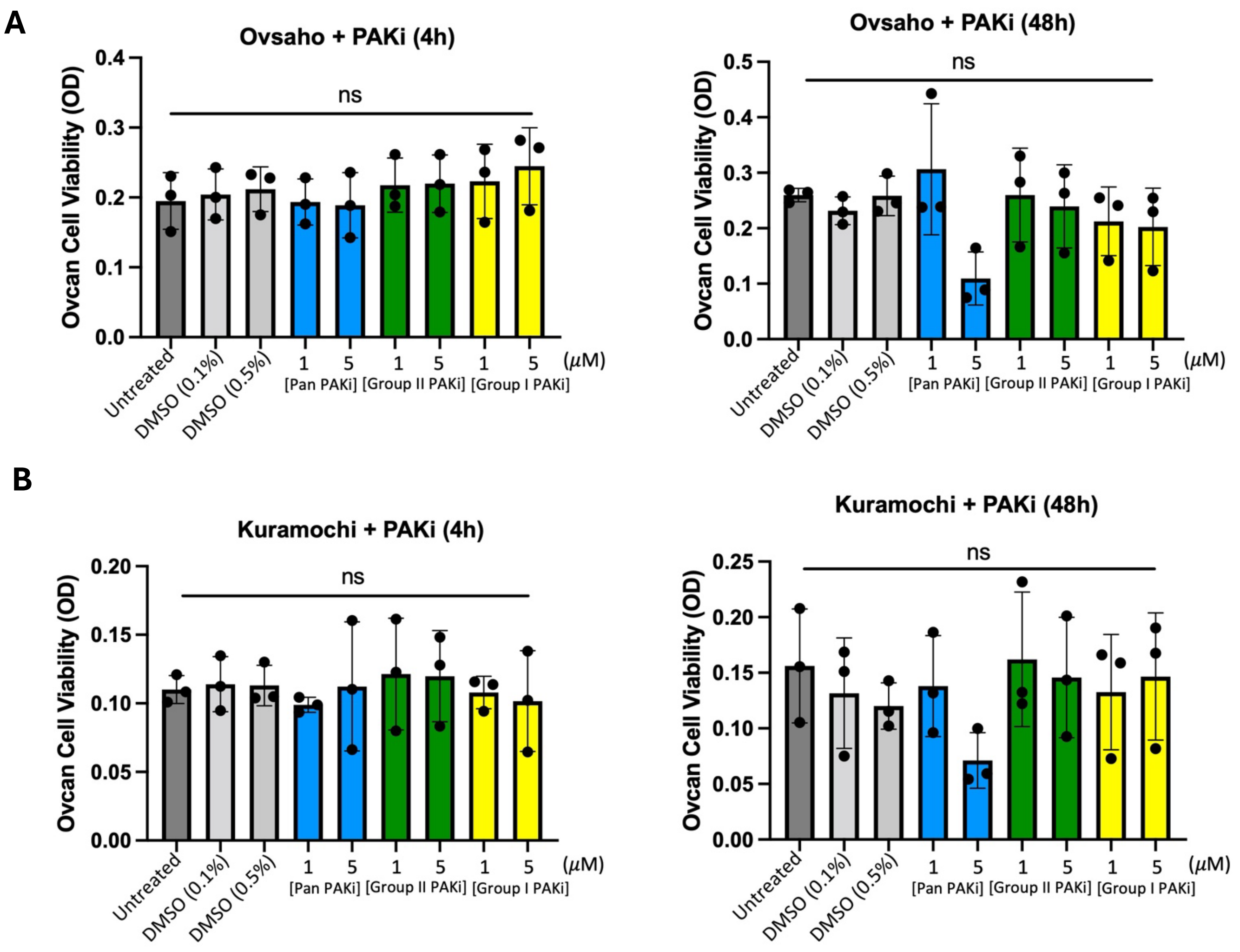

Supplementary Figure 4. Effect of PAKi on HGSC cell viability. A MTT viability assay of Ovsaho cells after 4hr (*left*) and 48hr (*right*) PAKi treatment, presented as mean ± SD with Pan PAKi (*blue bars*), Group II PAKi (*green bars*), and Group I PAKi (*yellow bars*) results. Statistical significance was calculated with one-way ANOVA. Plots show no significant difference in Ovsaho cell viability compared to untreated control after 4hr and 48hr PAKi. **B** MTT viability assay of Kuramochi cells after 4hr (*left*) and 48hr (*right*) PAKi treatment, presented as mean ± SD with Pan PAKi (*blue bars*), Group II PAKi (*green bars*), and Group I PAKi (*yellow bars*) results. Statistical significance was calculated with one-way ANOVA. Plots show no significant difference in Kuramochi cell viability compared to untreated control after 4hr and 48hr PAKi. All figures represent N=3 biologically independent experiments.

A

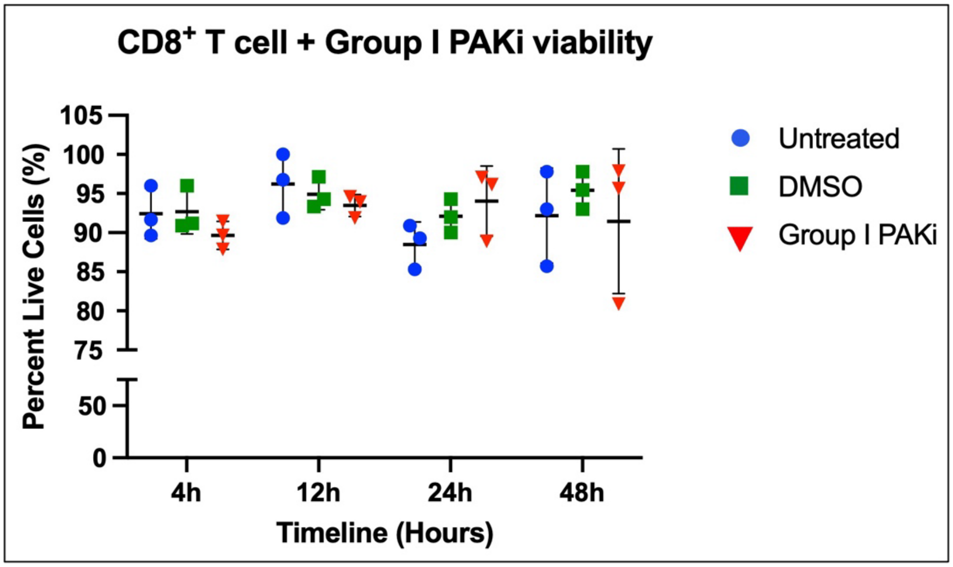

B

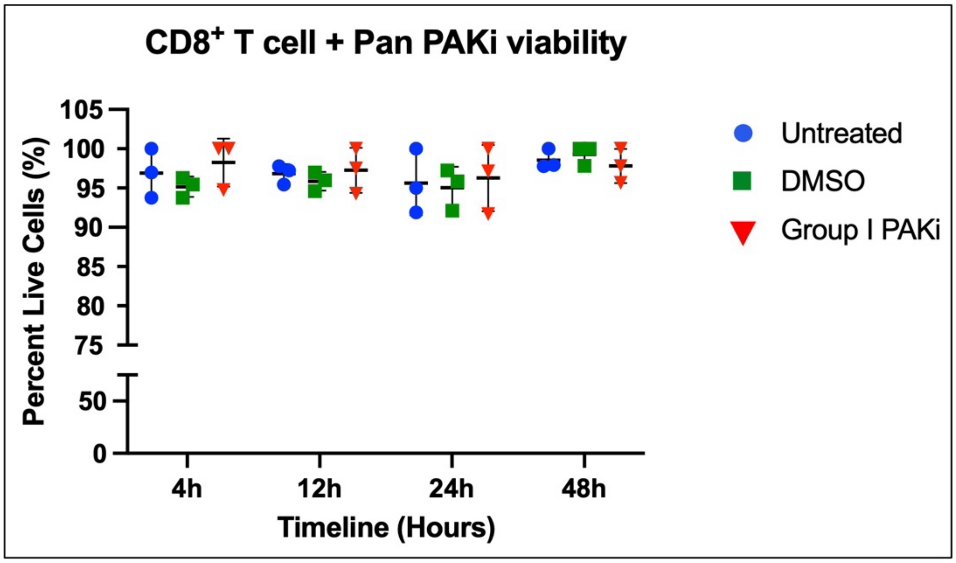

Supplementary Figure 5. Effect of PAKi on CD8⁺ T cell viability. A Plot shows ratio of live versus total CD8⁺ T cells after time course of Group I PAKi (5μM) treatment. Data are presented as mean ± SD with untreated (*blue circles*), DMSO (*green squares*), or Group I PAKi (*red triangles*) results. **B** Plot shows ratio of live versus total CD8⁺ T cells after time course of Pan PAKi (5μM) treatment. Data are presented as mean ± SD with untreated (*blue circles*), DMSO (*green squares*), or Pan PAKi (*red triangles*) results. Both figures represent N=3 biologically independent experiments.

A

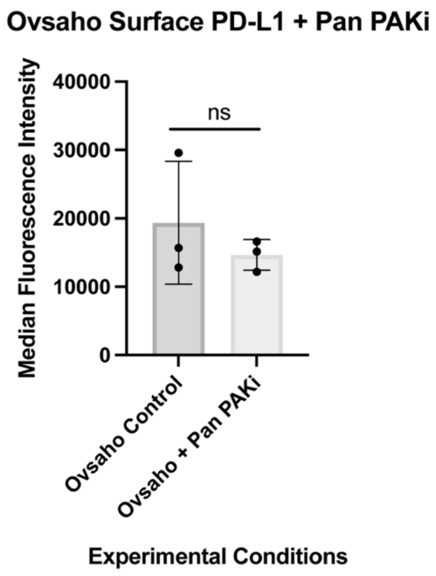

B

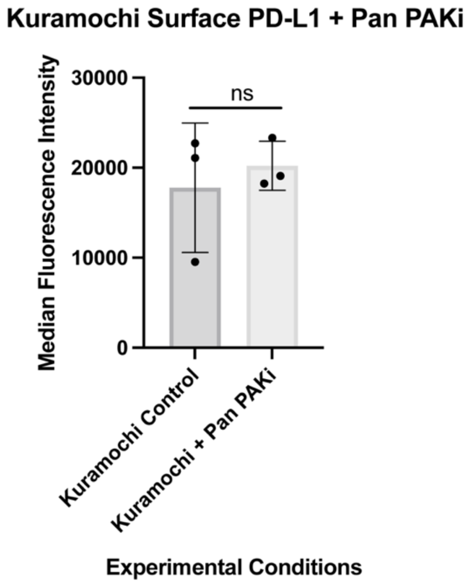

Supplementary Figure 6. Surface level expression of PD-L1 is maintained after PAKi in HGSC cells. Plots show quantification of median fluorescence intensity values of histogram overlays showing surface PD-L1 expression of Ovsaho **(A)** and **(B)** Kuramochi cells after 48hr Pan PAKi treatment. Data are presented as mean ± SD. Statistical significance was calculated with a student's t-test. Plots show no significant difference between untreated HGSC surface PD-L1 and Pan PAKi HGSC surface PD-L1 after 48h. Both figures represent N=3 biologically independent experiments.

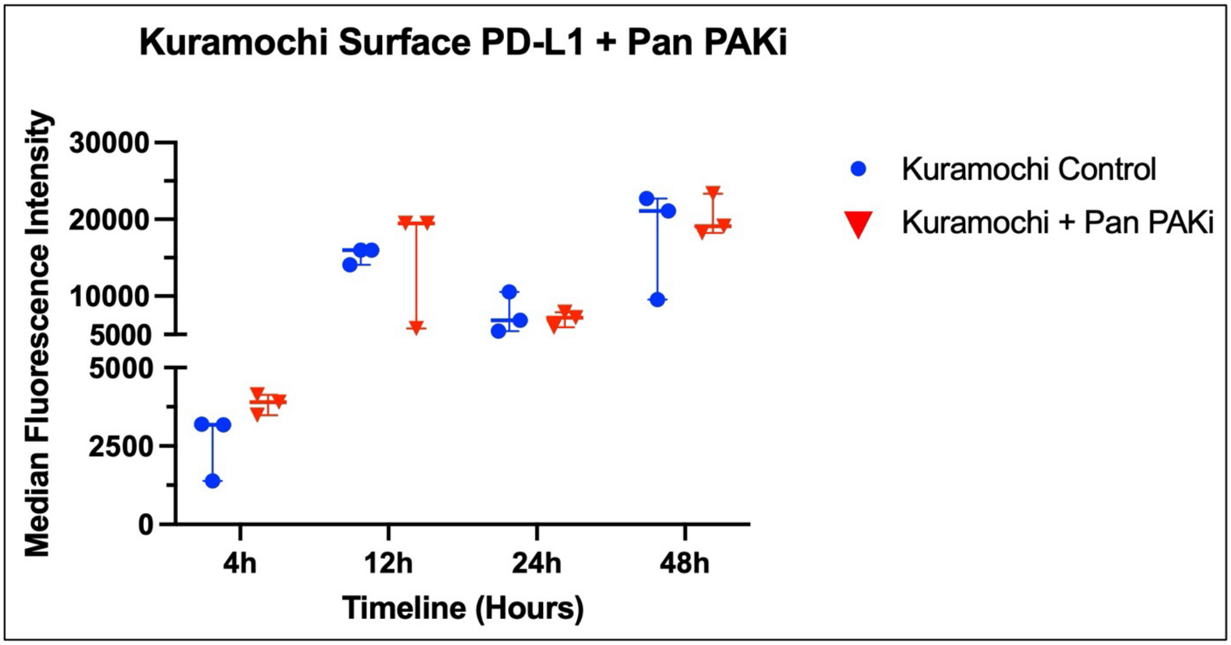

Supplementary Figure 7. Time course of surface PD-L1 expression following PAK inhibition. Graph shows surface PD-L1 expression of Kuramochi cells after a time course of Pan PAKi treatment (5μM), with median fluorescence intensity values plotted for untreated (blue circles) or Pan PAKi (red triangles) conditions. Data are presented as median ± range and represent N=3 biologically independent experiments.

A

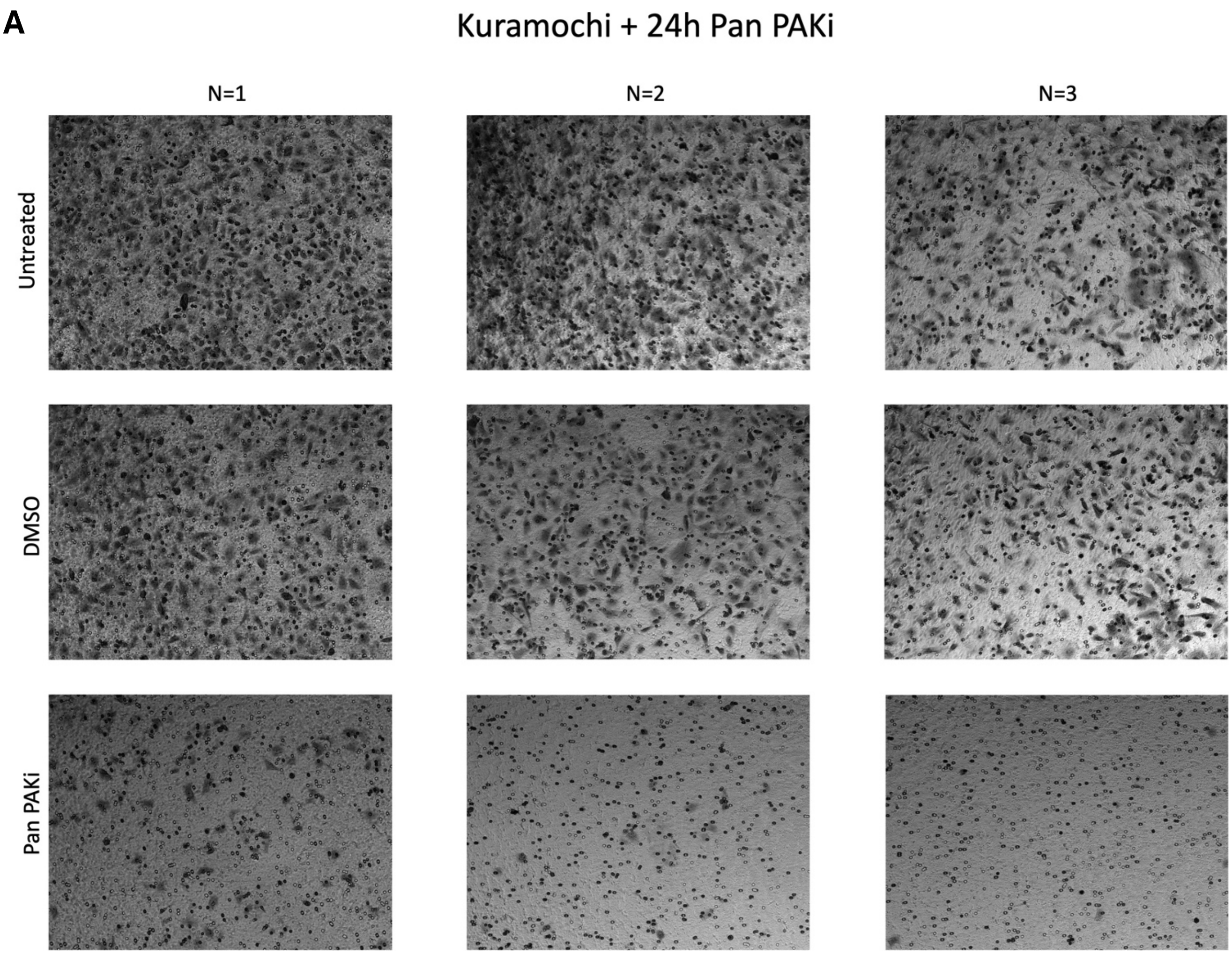

B

Supplementary Figure 8. Transwell Migration of HGSC cells after PAK inhibition. A Images show 24h transwell migration of Kuramochi cells in untreated, DMSO, and Pan PAKi (5μM) treated conditions after 6h serum-starvation. **B** Plot shows quantification of Kuramochi cell migration. Data are presented as mean ± SD and statistical significance was calculated with one-way ANOVA and post-hoc analyses. Results show a significant decrease in 24h Kuramochi cell migration with Pan PAKi and represent N=3 biologically independent experiments.

Ovsaho				
	Concurrent Treatment		Pre-Treatment	
(1μM)	% of Ovsaho remaining	% Killing	% of Ovsaho remaining	% Killing
Naive CD8 (1:4)	102.96	-2.96	79.34	20.66
Activated CD8 (1:4)	88.32	11.68	74.34	25.66
Pan PAKi	134.33	-34.33	103.58	-3.58
Act. + Pan PAKi [1:4]	113.58	-13.58	89.69	10.31
Group II PAKi	109.06	-9.06	77.69	22.31
Act. + Group II PAKi [1:4]	83.94	16.06	79.18	20.82
Group I PAKi	97.39	2.61	70.02	29.98
Act. + Group I PAKi [1:4]	72.66	27.34	56.24	43.76
(5μM)				
Naive CD8 (1:4)	102.96	-2.96	79.34	20.66
Activated CD8 (1:4)	88.32	11.68	74.34	25.66
Pan PAKi	82.61	17.39	59.04	40.96
Act. + Pan PAKi [1:4]	91.05	8.95	47.60	52.40
Group II PAKi	93.88	6.12	81.42	18.58
Act. + Group II PAKi [1:4]	75.67	24.33	51.49	48.51
Group I PAKi	89.20	10.80	67.52	32.48
Act. + Group I PAKi [1:4]	68.37	31.63	39.02	60.98

Supplementary Table 1. Concurrent and Pre-Treatment cytotoxicity assays of Ovsaho cells with PAKi and PBMC-derived CD8$^+$ T cells. Table results show increased killing [decreased viability] by activated PBMC-derived CD8$^+$ T cells when Ovsaho cells are pre-treated with PAKi compared to activated CD8$^+$ T cell killing after concurrent PAKi**.**

Kuramochi				
	Concurrent Treatment		Pre-Treatment	
(1μM)	% of Kuramochi remaining	% Killing	% of Kuramochi remaining	% Killing
Naive CD8 (1:4)	92.62	7.38	106.37	-6.37
Activated CD8 (1:4)	69.53	30.47	87.78	12.22
Pan PAKi	154.90	-54.90	162.53	-62.53
Act. + Pan PAKi [1:4]	53.35	46.65	75.50	24.50
Group II PAKi	143.38	-43.38	157.06	-57.06
Act. + Group II PAKi [1:4]	67.79	32.21	57.60	42.40
Group I PAKi	95.69	4.31	122.60	-22.60
Act. + Group I PAKi [1:4]	72.34	27.66	44.96	55.04
(5μM)				
Naive CD8 (1:4)	92.62	7.38	106.37	-6.37
Activated CD8 (1:4)	69.53	30.47	87.78	12.22
Pan PAKi	83.85	16.15	71.07	28.93
Act. + Pan PAKi [1:4]	58.87	41.13	45.10	54.90
Group II PAKi	85.78	14.22	55.40	44.60
Act. + Group II PAKi [1:4]	56.31	43.69	47.10	52.90
Group I PAKi	88.26	11.74	109.62	-9.62
Act. + Group I PAKi [1:4]	71.57	28.43	41.97	58.03

Supplementary Table 2. Concurrent and Pre-Treatment cytotoxicity assays of Kuramochi cells with PAKi and PBMC-derived CD8$^+$ T cells. Table results show increased killing [decreased viability] by activated PBMC-derived CD8$^+$ T cells when Kuramochi cells are pre-treated with PAKi compared to activated CD8$^+$ T cell killing after concurrent PAKi.

Ovcan Combination Therapy				
	Ovsaho		Kuramochi	
(1μM)	% of Ovsaho remaining	% Killing	% of Kuramochi remaining	% Killing
Activated CD8 (1:4)	81.52	18.48	85.89	14.11
Act. (1:4) + αPD-1	88.03	11.97	92.30	7.70
Pan PAKi	122.56	-22.56	99.50	0.50
Pan PAKi + Act. (1:4) +αPD-1	91.20	8.80	80.92	19.08
Group II PAKi	102.41	-2.41	95.04	4.96
Group II PAKi + Act. (1:4) +αPD-1	88.25	11.75	81.62	18.38
Group I PAKi	94.45	5.55	86.33	13.67
Group I PAKi + Act. (1:4) +αPD-1	82.49	17.51	84.85	15.15
(5μM)				
Activated CD8 (1:4)	81.52	18.48	85.89	14.11
Act. (1:4) + αPD-1	88.03	11.97	92.30	7.70
Pan PAKi	48.07	51.93	44.35	55.65
Pan PAKi + Act. (1:4) +αPD-1	30.59	69.41	23.90	76.10
Group II PAKi	97.04	2.96	76.14	23.86
Group II PAKi + Act. (1:4) +αPD-1	82.37	17.63	69.71	30.29
Group I PAKi	63.81	36.19	63.48	36.52
Group I PAKi + Act. (1:4) +αPD-1	54.32	45.68	59.29	40.71

Supplementary Table 3. Combination Therapy of PAKi and PBMC-derived CD8$^+$ T cells + αPD-1 in HGSC cells. Table results show increased killing [decreased viability] of HGSC cells by activated PBMC-derived CD8$^+$ T cells after PAKi pre-treatment and αPD-1.
